# The Acoustic Environment and University Students’ Satisfaction with the Online Education Method during the COVID-19 Lockdown

**DOI:** 10.3390/ijerph20010709

**Published:** 2022-12-30

**Authors:** Virginia Puyana-Romero, Angela María Díaz-Márquez, Giuseppe Ciaburro, Ricardo Hernández-Molina

**Affiliations:** 1 Department of Sound and Acoustic Engineering, Universidad de Las Américas, Quito EC170125, Ecuador; 2 Laboratory of Acoustic Engineering, Universidad de Cádiz, 11510 Puerto Real, Spain; 3 Place, Environment and Society Research Group, Universidad de Las Américas, Quito EC170125, Ecuador; 4 Department of Architecture and Industrial Design, Università degli Studi della Campania Luigi Vanvitelli, Borgo San Lorenzo, 81031 Aversa, Italy

**Keywords:** online education, indoor acoustic environment, students’ satisfaction, indoor soundscapes

## Abstract

The acoustic environment has been pointed out as a possible distractor during student activities in the online academic modality; however, it has not been specifically studied, nor has it been studied in relation to parameters frequently used in academic-quality evaluations. The objective of this study is to characterize the acoustic environment and relate it to students’ satisfaction with the online learning modality. For that, three artificial neural networks were calculated, using as target variables the students’ satisfaction and the noise interference with autonomous and synchronous activities, using acoustic variables as predictors. The data were obtained during the COVID-19 lockdown, through an online survey addressed to the students of the Universidad de Las Américas (Quito, Ecuador). Results show that the noise interference with comprehensive reading or with making exams and that the frequency of noises, which made the students lose track of the lesson, were relevant factors for students’ satisfaction. The perceived loudness also had a remarkable influence on engaging in autonomous and synchronous activities. The performance of the models on students’ satisfaction and on the noise interference with autonomous and synchronous activities was satisfactory given that it was built only with acoustic variables, with correlation coefficients of 0.567, 0.853, and 0.865, respectively.

## 1. Introduction

Distance education can be defined as an educational modality that “provides access to learning when the information source and the learners are separated by time and distance, or both” [1]. As early as 1728, there is evidence of the first distance education initiatives in the *Boston Gazette* of New England [2,3]; since then, technological advances, marked by the development of postal services (c. XVIII) and the emergence of electronic media (c. XX) [4,5] have favoured the evolution of distance education. However, it was not until 1969 that the British Open University became the first academic institution in the world to award university degrees for distance education, offering fully developed programmes based on new media and systematic assessment [2,6]. The first university to offer this type of study in Latin America was the Universidad Técnica Particular of Loja (Ecuador) in 1976 [7,8]. Subsequent advances in information and communication technology (ICT) led to the foundation in 1995 of the Universitat Oberta of Catalunya (Spain), which became the world’s first online university.

The space-time dynamics of online education allow the development of educational practices through digital resources, which have added specific didactic elements to university learning, such as non-face-to-face contact between teachers and students, or space and time flexibility to study and teach. However, there are many aspects of the environment that surround the student in online education that differ greatly from that of traditional classrooms, such as the acoustic environment. ISO 12913 defines the acoustic environment as the “sound at the receiver from all sound sources as modified by the environment” [9].

The World Health Organization (WHO) recommends that in order to hear and understand messages broadcasted in educational settings, the background noise level should not exceed 35 dBA [10]. Various studies conclude that in most classrooms designed specifically for teaching, the average background noise level exceeds the WHO recommendations and therefore compromises the ability of students to listen to the teacher [11,12]. This effect is probably more accentuated at home, where there are numerous noise sources that far exceed the WHO recommended noise levels (e.g., TV, radio, or household appliances).

The exposure to high noise levels affects not only students’ learning [13] and reading ability [14,15] but also their motivation [16,17] and their degree of annoyance [18]. Combined effects of different noise sources to which students have been exposed throughout their lives (e.g., noisy toys, video games, or sound reproduction equipment) may damage their auditory system, making them susceptible to experiencing learning problems. In fact, a recent study shows that more than 40% of adolescents report tinnitus (a symptom of damage to the peripheral auditory system, for which there is currently no cure), so their ability to understand acoustic messages is reduced [19]. These acoustic problems especially affect those with some type of adaptation problem, with hearing difficulties or with a native language that does not coincide with that of their place of residence [20,21].

During the COVID-19 lockdown, the online learning situation had nuances of a different nature than those of the traditional online modality [22]. Furthermore, the students had not chosen this type of education, but rather, it was imposed by lockdown circumstances. Therefore, some students had to attend lessons in places not adapted for learning and with unsuitable means. Synchronous activities led to simultaneity problems (e.g., the internet network or shared rooms) when there was more than one student at home or when other family members worked online. In most cases, synchronous activities occurred while all the family members (younger siblings, parents, and elderly dependents) were at home engaging in different activities, leading to distractions that even further hinder the learning processes. Fluidity issues of the internet service and the massive connections at the same time also led to difficulties in connectivity that made communication difficult. Most of the mentioned situations created high background noise that did not contribute to an appropriate acoustic environment for developing academic activities or affected the communication channels between student and teacher, in which sound plays an important role.

Given the growing interest in online learning, many organizations have taken advantage of these months of forced confinement to assess the degree of student satisfaction with this learning system, intending to launch future platforms that follow this modality. This concern is not new, in that numerous studies have evaluated the influence of different factors on the degree of satisfaction with online teaching [23,24,25,26]. Course design and structure [27], subject matter [28], learner–content interaction (LCI), learner–instructor interaction (LII) [24], workload, technological support, pedagogical skills [29,30], and a set of “achievement emotions” [25] are some of the many proposed factors found in the literature concerning student satisfaction. Hence, the conceptualization of student satisfaction has been studied mainly at the interface between the student and the system that provides the online service. The impact of noise sources in the student surroundings is not well explored as a relevant factor in such research; however, notions concerning students’ experiences, such as concentration challenges [31], obstacles to completing tasks [32], and distractions [33,34], among others [33,35], show recurrent mentions of environmental noise as a common interfering disturbance.

In recent times, more and more researchers have been addressing the prediction of variables using a data-driven approach. The machine-learning-based algorithms allow us to guide the simulation and the decision-making process by using the data collected in survey campaigns. In this context, artificial neural networks (ANNs) offer the tools to extract knowledge useful for different disciplines; for instance, in acoustics, they have been used to remove noise from speech signals [36], to categorize the outdoor acoustic environment [37,38], and to predict urban noise [39] or soundscape conditions [40]. In the education field, they have been used to predict students’ performance [41] or their degree of satisfaction [42] with the online learning modality. However, studies that apply ANNs to predict students’ satisfaction with online academic environments on the basis of the acoustic environment at home have not been found.

**Figure 1:**
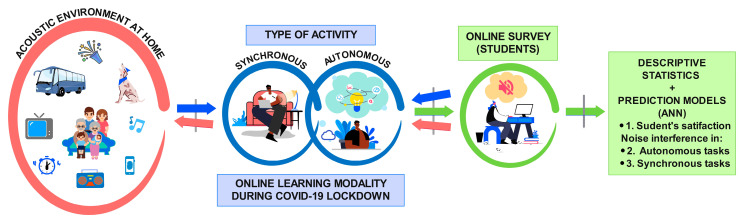
Conceptual scheme of the workflow followed in the present research.

The objective of this study was to evaluate the influence of the acoustic environment at home on students’ satisfaction with the online learning modality. For that, three artificial neural networks models were calculated by using perceived acoustic factors as explanatory variables. The first model was developed to predict students’ satisfaction with the online learning modality. The other two models were calculated to measure the role of the acoustic variables on the interference with synchronous activities (those activities that occur when students and teachers interact in real time and at the same time; consequently, both must meet online) and autonomous activities (those that students carry out by their own means, without simultaneous interaction with the teaching staff) (Figure 1). The data were collected during the period of confinement owing to the COVID-19 disease, through a survey addressed to the students of the Universidad de Las Américas in Quito, Ecuador.

## 2. Materials and Methods

To evaluate the influence of the acoustic environment at the students’ homes, an online survey was carried out. Subsequently, the following data analyses were performed: descriptive statistics, features selection methods, ANNs, and the relative importance of the variables used for the ANNs.

### 2.1. Online Survey: Description and Questionnaire Design

The online survey was carried out during part of January and February 2021 and was open to the students’ participation for 20 days. The questionnaire was developed in Microsoft Forms and could be responded to only by the students of the Universidad de Las Américas, in Quito, Ecuador, as only they had access to the inner university network of Microsoft. The questionnaire was addressed to students enrolled in undergraduate or master’s courses of all the academic disciplines offered by the Universidad de Las Américas. Several emails were sent requesting the students’ participation with a link that gave them access to the survey. Once started, the student could go on with the questionnaire only if they had answered all the previous questions. The platform registered the responses of only those students who had finished the whole questionnaire. To guarantee the confidentiality of the participants, the answers to the survey were managed by the Department of Information Intelligence of the Universidad de Las Américas, which codified the responses of each participant through a number and hid their email addresses. A total of 2477 participants finished the survey.

The online survey contained general questions about personal data (age, gender, and current semester—the semester in which the survey was conducted), questions about the acoustic environment, and one question about students’ satisfaction with the online learning method (Q1). Except for age, which was open-ended, all the other questions used in the present study were closed, single-choice type. The question about age allowed only numeric input. Because the number of semesters of different disciplines differs and because students might take subjects from different levels, we divided the possible responses to the question about the current semester into three categories, namely initial, intermediate, and final; this allowed students to self-classify. Table A1 (Appendix A) summarizes the questions about students’ satisfaction and the acoustic environment at home with their possible responses; it also shows the correspondence between the question and the acronym of the variables analysed in the present study. The questions about the acoustic environment were related to the perceived loudness of the sound sources (Q2), the interference of the sound sources in autonomous (Q3) and synchronous activities (Q4), the frequency of issues during synchronous activities related to sounds (Q5), the noise interference in specific autonomous tasks (Q6), the noise’s provenance (Q7), the general noise interference in autonomous and synchronous tasks (Q8), the frequency of noise connection problems (Q9), the ability of the student to endure noise (Q10), and the order of interference with academic activities (from a list of 5 factors) within the acoustic environment (Q11).

The answers to the questions on the acoustic environment and students’ satisfaction were presented on Likert scales of 5, 6, and 7 points. For some questions, the activities covered by the survey were divided into synchronous and autonomous.

The exclusion criteria were the lack of response to any of the questions of the study (because of a possible web-platform issue), and an ambiguous response to the age question. According to these criteria, no responses of any participant were discarded.

### 2.2. Data Analysis

**Figure 2:**
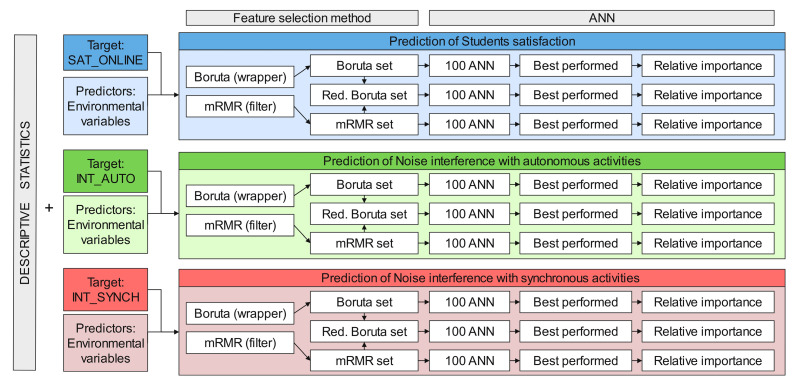
Workflow of the data analysis performed. The target variables of the prediction models are students’ satisfaction with the online learning method (SAT_ONLINE) and the noise interference with autonomous activities (INT_AUTO) and synchronous activities (INT_SYNCH).

The workflow of the statistical analysis is shown in Figure 2.

Descriptive statistical analysis was performed to characterize the sample and better understand the behaviour of the variables; in particular, the relative frequency and measures of central tendency (median and mode) and position were calculated. Age, gender, semester, and the target features (SAT_ONLINE, INT_SYNCH and INT_AUTO) were the variables used. As age was split into groups, the descriptive analysis was applied only to ordinal and nominal variables.

Although it might be thought that it is important to have as many variables as possible to calculate predictive models, we would risk using redundant data or data that do not provide relevant information, which can lead to a loss of model efficiency [43]. That is why feature-selection methods are frequently used before carrying out machine-learning-based models, for example to predict disease risk [44,45], wind speed [46], and soundscape quality [47] or to classify text [48] or images [49]. As we have a high number of explanatory variables, and in order to avoid the risks previously mentioned, two feature-selection methods were tested: (1) a filter method (minimum redundancy, maximum relevance—mRMR) and (2) a wrapper method (Boruta). The embedded methods were not tested, as the feature-selection algorithm is integrated as part of the learning algorithm [50], and we preferred to use ANN models in an independent step, to more easily control the network configuration and its output.

(1) The filter methods perform a selection of features independently of the learning algorithm that will be used. These methods carry out a classification utilizing a function that after evaluating each feature, assigns them a value that is used to order the variables and to set a threshold to eliminate those of less relevance [51]. Among the different filter models, we chose the mRMR method because it measures, using two algorithms, not only the mutual information between the variable and the response (“maximum relevance” algorithm) but also the mutual information between the predictors (“minimum redundancy” algorithm). Other reasons for choosing the mRMR method are its low computational time and its better generalizability compared with other feature-selection methods. However, control over the number of features selected depends on the settings stablished by the user. For this study, the mRMRe package of R was used [52].
(2) Wrapper methods, unlike filter methods, extract a subset of variables that obtain the best possible fit with a given algorithm [53,54]. Boruta is a wrapper method that uses random forest as the underlying algorithm. The method generates in each iteration a series of shadow variables from the predictors, copying each of them and permuting the elements of each new column with each other. In this way, the variables do not compete with each other but rather with a random version of them (shadow). Once the Boruta model has been fitted, the relative importance of each variable is calculated and three groups of variables are defined: confirmed important, confirmed unimportant, and tentative variables (those for which the algorithm was not able to arrive at a conclusion about their importance) [55]. The Boruta package of R Software [55,56] was chosen among other wrapper methods because it is capable of capturing nonlinear relationships between variables and allows for increasing the number of iterations to obtain better results. Another advantage of the model is that it indicates the most appropriate number of variables to get good performance from the prediction models. Among the drawbacks, it needs hyperparameter tuning to improve the results and consequently requires more computational time than filter methods do. It can also lead to overfitting the models [50,57].

For each feature-selection method, the target variables were SAT_ONLINE, INT_SYNCH and INT_AUTO (separately considered). For the SAT_ONLINE model, the explanatory data were the variables from the acoustic environment corresponding to questions 2 to 11 of Table A1. For both the INT_SYNCH and SYNCH_AUTO models, the explanatory variables corresponded to questions 2 to 7 and 9 to 11. The input data (ordinal variables) were standardized before applying the feature-selection methods to transform the variables measured on a common scale.

Artificial neural networks (ANNs) are mathematical models inspired by the biological behaviour of neurons and the structure of the brain. Simulating the behaviour of real neurons involves a high level of abstraction. In an ANN, there are input variables, which are multiplied by random values (weights); a mathematical function is applied to this set, which determines whether the neuron is activated. Subsequently, another function calculates the output of the neural network. Consequently, ANNs are composed of groups of input, hidden, and output layers [58,59]. The value of the weight that is initially assigned to the neuron determines the strength with which the neuron is computed. Iteratively adjusting the values of the weights, the output of the network is obtained, which must be an approximation of the target variable. This process of the iterative modification of the weights is called learning or training the network. In this study feedforward artificial neural network models (multilayer perceptron—MLP) were calculated using the R package “neuralnet” [60].

Three sets of input variables were used to calculate the ANN models for each target variable (SAT_ONLINE, INT_SYNCH and INT_AUTO). Two of these sets were selected by the mRMR and Boruta methods. As Boruta selected a high number of variables, an additional set was built with the same number of variables chosen by the mRMR but composed of the first variables of Boruta (in decreasing order of relative importance), in order to compare the results of both methods. Henceforth, this set of variables will be called “reduced Boruta”.

The relative importance of the input variables used for building the ANN was evaluated by applying the Olden et al. method [61], as it helps to understand the contribution of each variable in the conformation of the model.

## 3. Results

### 3.1. Descriptive statistics

The sample consisted of 1371 women (55.3%), 1103 men (44.5%), and 3 uncategorized-genre students (0.12%). The mean age was 21.9 years old (standard deviation ±4.2), with a minimum of 17 and a maximum of 68; 85.1% of the subjects were under 25 years old, 11.4% were between 25 and 30, and only 3.5% of the sample were over 30. The ‘current semester’ of most students (41.8%) corresponded to ‘intermediate’, while ‘initial’ and ‘final’ levels were pretty even at 29.9% and 28.3%, respectively.

**Figure 3:**
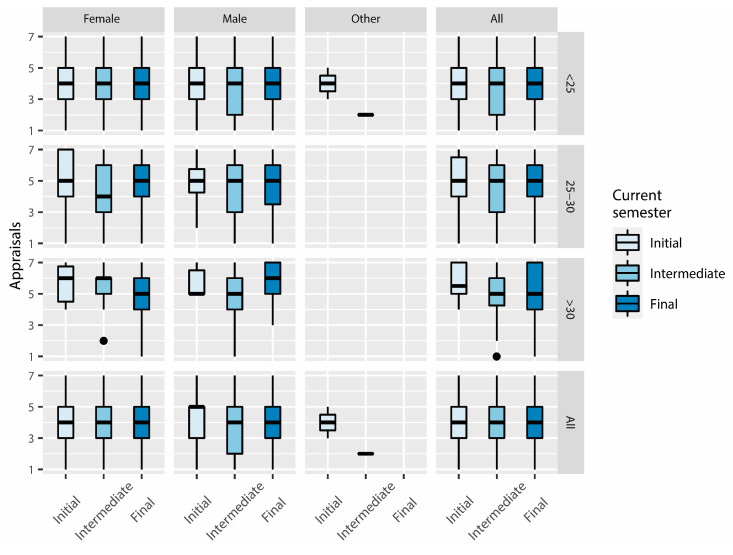
Box plot of the students’ satisfaction with the online learning method, split by ‘gender’ (‘Male’, ‘Female’, ‘Other’, and ‘All’), ‘age’ (‘<25′, ’25–30′, ‘>30′, and ‘All’), and ‘current semester’ (semester in which the survey was conducted).

Figure 3 shows the distribution of the appraisals given to the students’ satisfaction with the online learning method, within each ‘current semester’ (or semester in which the survey was conducted), split by ‘gender’ and ‘age’ ranks. The appraisals were given in a 7 points Likert’s scale (bipolar), from ‘1-very unsatisfied’ to ‘7-very satisfied’.

For the ‘Female-<25′ group, the first, second, and third quartiles are equal and do not depend on the semester that they were enrolled in, which is repeated when the whole ‘Female’ group is considered; furthermore, the scores present a symmetric distribution. For the ‘Initial-Female-25–30′ and ‘Intermediate-Female-25–30′ subgroups, however, the distributions are positively skewed. According to the median values, and evaluating the percentages, more female students between 25 and 30 are satisfied than indifferent or unsatisfied. Nearly the same is true for the ‘Female->30′ group, in which median values are ≥5 for all the semesters.

The median scores of the ‘Male’ groups ≥ 25 years old are ≥5, which means that most students are satisfied with the online learning method (which is the same in the female groups for the same ages).

The satisfaction scores of ‘All’ the students under 25 years old show a similar distribution for the ‘Initial’ and ‘Final’ semesters: the first and third quartiles have 3 and 5 respectively. The interquartile range was IQR = 2. For the ‘Intermediate < 25′ students, however, the distribution is negatively skewed. This trend of the ‘Intermediate-<25′ group seems to be marked by the scores of the ‘Male-Intermediate-<25′ subgroup, which shows the same distribution. The median scores of ‘All’ the students ≥25 years old are ≥5, which is the same for male participants ≥ 25.

When evaluating the group ‘All-All’ (without splitting by gender or age), we can appreciate a symmetric distribution, following the trend of the ‘Female-All’ group.

The evaluation of the percentages of responses of each semester, for all ages and genders, indicates that ‘Satisfaction’ (scores > 4) is slightly higher for the ‘Initial’ and ‘Final’ categories, whereas the percentage of ‘Unsatisfied’ (scores < 4) and ‘Satisfied’ students in the ‘Intermediate’ level is very similar (the difference is merely 0.04%).

**Figure 4:**
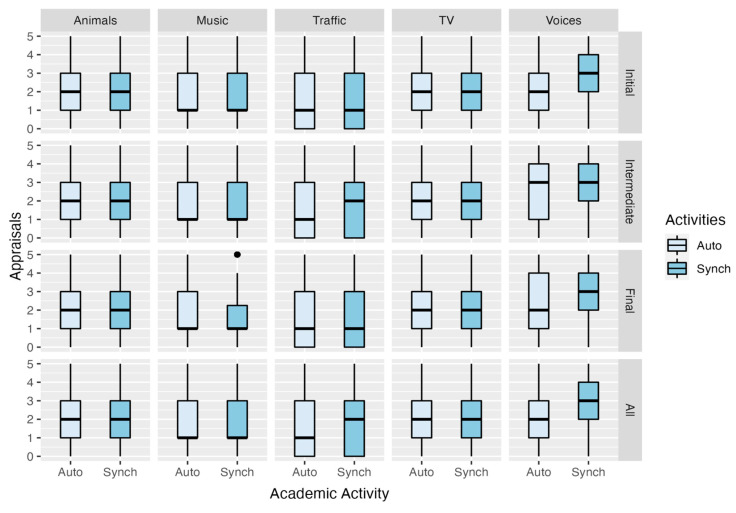
Box plot of the responses given to the degree of interference from different noise sources (‘Animals’, ‘Music’, ‘Traffic’, ‘TV/Radio/Household Appliances’, and ‘Voices’) with regard to students’ engaging in autonomous (Auto) and synchronous (Synch) activities, split by the ‘current semester’ (semester in which the survey was conducted).

Figure 4 displays the distribution of the appraisals given to the degree of interference of five noise sources (‘Animals’, ‘Music’, ‘Traffic’, ‘TV/Radio/Household Appliances’, and ‘Voices’) over autonomous and synchronous activities, according to the ‘kind of noise sources’ and the ‘current semester’. The responses were given on a 6-point Likert scale (unipolar), where ‘0-I did not hear them’, ‘1-They did not interfere’, and ‘5-Extremely’. We should emphasize the highly similar distributions between both types of academic activities and among all the ‘current semester’ categories; for example, the distributions on the degree of interferences in autonomous and synchronous activities are the same for the sound sources ‘Animals’ and ‘TV/Radio/Household Appliances’. Hence, for the whole sample of these variables, the scores vary with an interquartile range, IQR = 2, between appraisals 1 and 3 with a median of 2.

For music as a source of interference, the plot shows that scores vary in the same manner for all categories of the ‘current semester’ and each of the academic activities, with an IQR = 2—except for the subgroup ‘Music-Final-Synch’, which exhibits less variance (IQR = 1.2). However, in all cases, the distribution is positively skewed, with median and first quartile in the noninterference scores.

The perceived interference of ‘Traffic’ during academic activities spread more widely within the scale than the previous variables did, for all subgroups under examination (IQR = 3). For that noise source, positive skewness is evident in the group ‘Traffic-Auto’ for all the semesters, mainly because the percentage of scores with the minimum possible valuation (0-I did not hear them’) is at least 25%. For instance, the proportion of 0 scores within the whole sample is 35.8% for autonomous activities and 31.0% for synchronous activities, and the situation is similar for all categories of ‘current semester’. Finally, although the first quartile is equal to the minimum score, the central tendency for the subgroup ‘Traffic-Intermediate-Synch’ is higher (with median = 2) than that of the rest (median = 1); this distribution can also be observed when factoring in the whole sample.

### 3.2. Minimum Redundancy Maximum Relevance Feature-Selection Method

The topology of the ensemble tree of the mRMR feature-selection method was set with 4 levels and 2 children for each element. For that, a vector of type integer containing the number of children of each element at each level of the resulting network tree is generated. The combined configuration of the levels and children affects the number of variables selected by the mRMR method. These settings were applied to the three dependent variables under study.

The variables selected for each model have been included in Table 1, together with the score calculated with the mRMR package. The score is the mutual information between the target and the predictor evaluated, minus the average mutual information of the previously selected predictors and this predictor. Consequently, negative scores mean that the average mutual information of the evaluated predictor with other predictors is higher than the mutual information with the target. The output of the mRMR was composed of 7 independent variables for the model of students’ satisfaction, 8 for the model of the noise interference with autonomous activities, and 6 for the model of the interference with synchronous activities.

Common selected features for the models of INT_AUTO and INT_SYNCH: the interference of noise coming from inside (N_INSIDE; Score_Auto = 0.3398, Score_Synch = 0.3906) and outside (N_OUTSIDE; Score_Auto = 0.1791, Score_Synch = 0.1685) are features related to the provenance of the noise, selected for both models. This was the same for the perceived loudness of voices (LOUD_VOI; Score_Auto = 0.2408, Score_Synch = 0.2644), TV/radio/home appliances (LOUD_TV; Score_Auto = 0.1885, Score_Synch = 0.1836), and traffic (LOUD_TRA; Score_Auto = 0.0443, Score_Synch = 0.0436).

Features selected only for the model INT_AUTO (and not for INT_SYNCH): the perceived loudness of animal noises (LOUD_ANI; Score_Auto = 0.0289) was an additional loudness variable selected only for this model. Regarding the interference with specific tasks during autonomous activities, writing essays (AUTO_WRI; Score_Auto = 0.0295) and taking exams (AUTO_EXA; Score_Auto = 0.0518) were also selected. It is remarkable that among 30 variables, the mRMR method (for the model of noise interference with autonomous activities) did not select any variable specifically linked to synchronous tasks.

Features selected only for the model INT_SYNCH: regarding the interference with specific tasks during synchronous activities, only losing track of the class (SYNCH_THR; Score_Synch = 0.1956) was selected. Similar to what happened with the variables selected for the model of autonomous activities, only variables related to synchronous tasks were selected.

Features selected for the model SAT_ONLINE: variables related to noise interfering with engaging in both autonomous activities (SYNCH_THR; Score_Satisfaction = 0.0600, and SYNCH_ATT; Score_Satisfaction = 0.0514) and synchronous activities were selected (AUTO_EXA; Score_Satisfaction = 0.0507, and AUTO_COM; Score_Satisfaction = 0.0513). Three more variables were chosen by the mRMR method, the frequency of audio problems caused by an internet connection (INTER_AUDIO; Score_Satisfaction = −0.0001), the ability of the students to endure a noisy environment (ENDURE_NOI; Score_Satisfaction = −0.0007), and the order given to the influence of noise on interfering with students’ engaging in academic activities (ORDER_IN_2; Score_Satisfaction = 0.0010) from a list of different environmental conditions (thermal, illumination, etc.).

**Figure 5:**
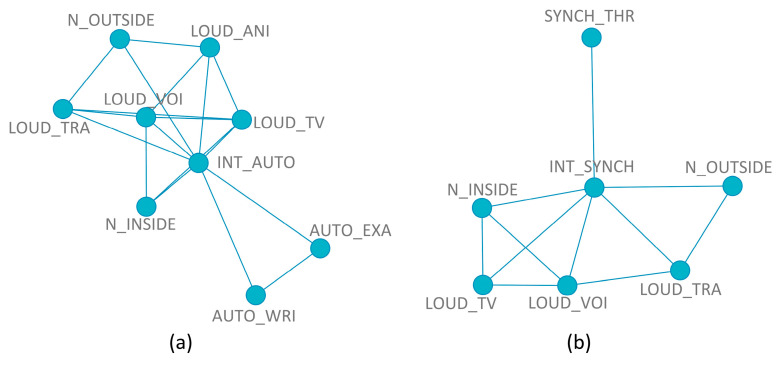
Inferred mRMRe network topology for the noise interference with autonomous (**a**) and synchronous (**b**) activities.

The inferred mRMR network topologies considering the autonomous (a) and synchronous activities (b) as dependent variables are shown in Figure 5. The coherence of the variables selected among them and with the dependent variables is remarkable.

For noise interference with autonomous activities, the noise outside (N_OUTSIDE) is related only to the perceived loudness of animals (LOUD_ANI) and traffic noise (LOUD_TRA), while the perceived loudness of voices (LOUD_VOI) and TV/radio/household appliances (LOUD_TV) is linked to the noise inside (N_INSIDE). There is also an association between the perceived loudness of animals, voices, and TV/radio/household appliances and between the perceived loudness of traffic and voices. Taking exams (AUTO_EXA) and writing essays (AUTO_WRI) are in a separate group; the only links are between them and with the dependent variable.

Regarding the selection of the input variables for the model of synchronous activities, similar responses to the noise inside/outside and the interference from the noise sources can be found (N_OUTSIDE is associated only with LOUD_TRA; N_INSIDE is linked to LOUD_TV and LOUD_VOI, and LOUD_VOI is linked to LOUD_TRA); the same can be found with the tasks that are interfered with by noise during synchronous activities (SYNCH_THR is isolated).

**Figure 6:**
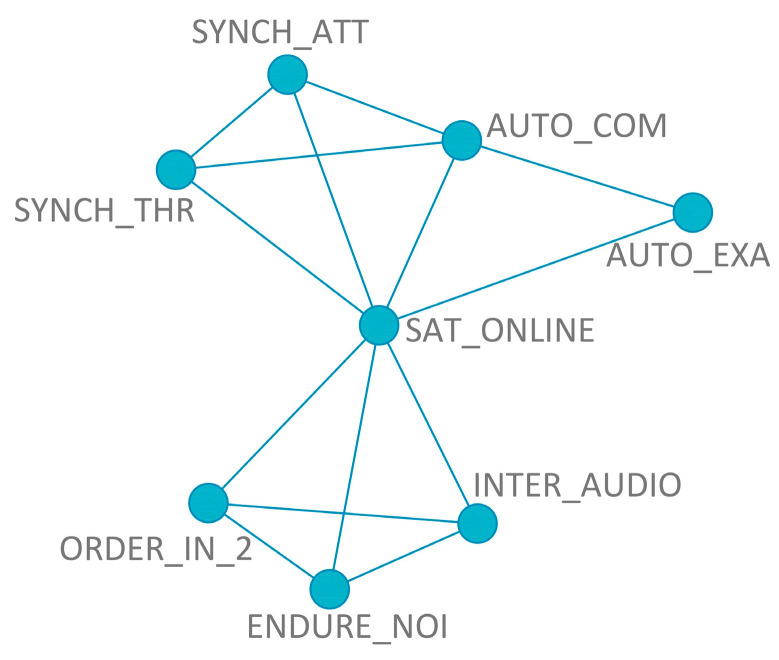
Inferred mRMRe network topology of the selected variables for the model of students’ satisfaction with the online learning method.

The network topology of Figure 6, for the model of students’ satisfaction, shows that there is a balance in the autonomous and synchronous activities, as two of each were selected by the mRMR method (SYNCH_THR, SYNCH_ATT, AUTO_COM, and AUTO_EXA). The audio quality of the internet (INTER_AUDIO), how much the students would endure noise (ENDURE NOISE), and the order of noise interference with a comparison between other activities (ORDER IN_2) were in a different group, with links among them (and with the dependent variable), but without links to the other variables.

### 3.3. Boruta Feature-Selection Method

The Boruta feature-selection method was calculated to compare the results with the ones of the mRMR algorithm. Boruta allows for setting up the *p*-values and the maximum number of iterations of the algorithm. The higher number of iterations, the more selective the algorithm becomes in picking variables. The default *p*-values (‘*p*-value’ = 0.01) and the number of times the algorithm is run (‘maxRuns’ = 100) were used. In the process of deciding whether a variable has been confirmed as important, some variables may be classified as ‘Tentative’. To guarantee that the tentative variables are being correctly excluded, the ‘TentativeRoughFix’ function was used [56].

**Figure 7:**
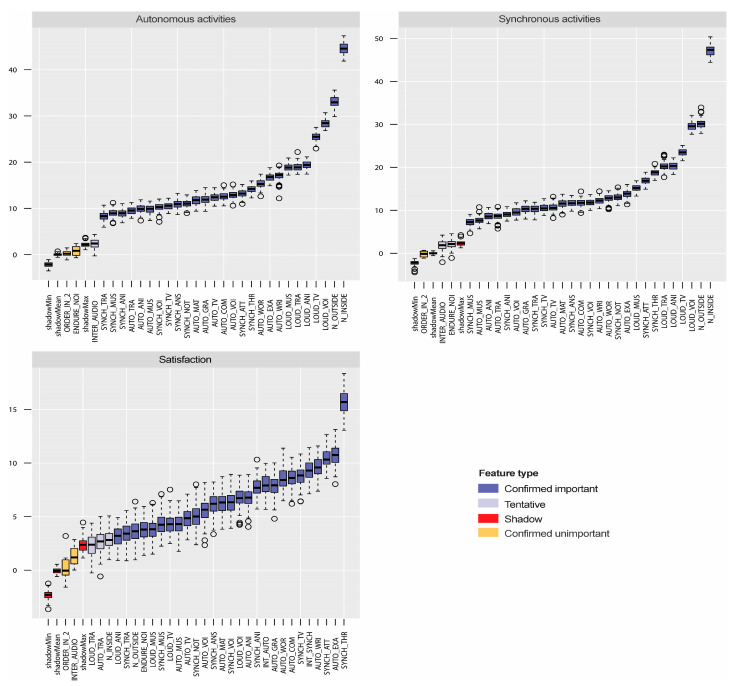
Relative importance of the attributes selected by the Boruta feature-selection method for the three dependent variables under study, students’ satisfaction with the online learning method (Satisfaction), and the noise interference with the autonomous and synchronous activities.

Figure 7 shows, through box plots, the relative importance of the variables selected by the Boruta algorithm for the models of students’ satisfaction with the learning method and the models of the noise interference with the autonomous and synchronous tasks (after applying the ‘TentativeRoughFix’ function). Blue box plots represent the variables selected by Boruta as important for the conformation of the model, and orange box plots are confirmed to be unimportant. The red box plot corresponds to the shadow attributes. There are three red box plots for the minimum (shadowMin), mean (shadowMean), and maximum (shadowMax) shadow attributes. The three models selected 27 variables. It is remarkable that for the model of satisfaction, the Boruta algorithm excludes variables that instead were selected by the mRMR method (ORDER_IN_2 and INTER_AUDIO).

### 3.4. ANN Models

To train each ANN, the resilient backpropagation algorithm with backtracking was used. Resilient backpropagation is an adaptive learning algorithm that iteratively modifies the weights of a neural network to minimize the error, keeping away from the local minimum and providing faster training than the backpropagation algorithm does [62]. The backtracking technique updates the weight values, reverting the previous iteration and adding a small rate to the weight in order to improve the performance of the network [63].

When working with an ANN, it is important to take the necessary measures to avoid overfitting. Overfitting happens when the learning model is capable of predicting only the cases used to teach the network but is unable to recognize new input data. To improve the generalizability of the model, the data can be divided into three sets, one for training and the other two for validating and testing the data. Another way to reduce overfitting is to avoid using hidden layers in excess; in this way, the model will be more flexible and capable of adapting the activations to the new entries [64,65]. To avoid overfitting, the data were divided into three sets chosen randomly: learning set (70%), validation set (15%), and test set (15%). Three artificial neural networks with three layers (1 input, 1 hidden, 1 output) were built for each dependent variable (SAT_ONLINE, INT_AUTO, and INT_SYNCH).

The root mean square error (RMSE) and the correlation coefficient were calculated to evaluate the performance of the ANN. The RMSE is normally used to explain how different the calculated values are from the measured values [66]. The RMSE is not scale invariant, and therefore its value may be affected by the scale of the data; for this reason, the data were standardized before calculating the ANN. The correlation coefficient is used to describe the relationship between two or more parameters on the basis of a straight-line model [67].

Subsequently, the Oldent et al. for the assessment of the relative importance of the input variables was calculated [68,69].

#### 3.4.1. ANN Model of Students’ Satisfaction with the Online Learning Method

Table 2 shows the three sets of input variables for the models of students’ satisfaction, listed in descending order of importance (according to the Boruta relative importance, in Figure 7, and the mRMR scores, in Table 1). Here, 100 ANN models with different input seeds and 10 iterations were run (for each set of input variables) to obtain the best-performing ones [47]. The model with the highest mean correlation coefficient calculated with the train, validation, and test sets was considered the best.

Table 3 shows the correlation coefficients and the RMSE for the train, validation, and test sets of the best-performing models to predict students’ satisfaction with the online learning method. The ANN calculated using Boruta-selected variables as predictors have a stronger mean correlation coefficient (r_Boruta_ = 0.5213) than the other models (Table 3), but also, the number of predictors is larger: 27 variables of the ANN–Boruta (from a total of 32), in comparison with 7 variables used for the other models (r_ReducedBoruta_ = 0.4586 and r_mRMR_ = 0.4698). No remarkable differences can be found in the correlation coefficients of the ANN models using mRMR or the set of variables of reduced Boruta. For the three models, the RMSE is similar for the train and test sets of data; however, for the validation, the RMSE is significantly lower in the model that used Boruta-selected variables.

**Figure 8:**
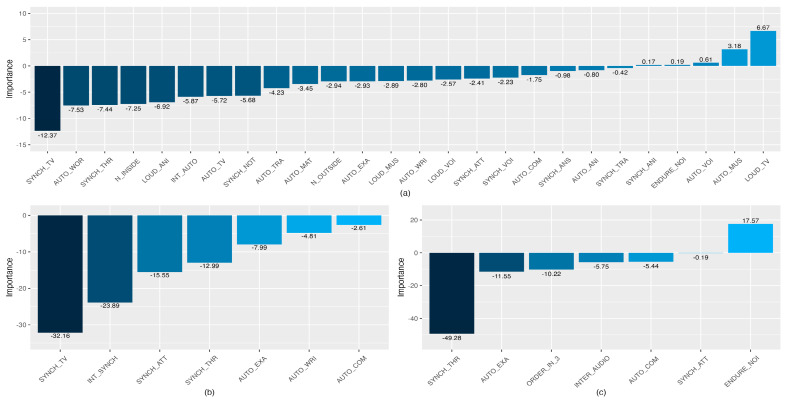
Percentage of the relative importance of the predictors of the ANNs on students’ satisfaction, calculated with the Olden et al. method [61]. The variables selected by Boruta (**a**), mRMR (**c**), and the set of reduced Boruta variables (**b**) were used as predictors. Values higher than zero mean positive relative contribution, and those lower than zero mean negative relative contribution.

The relative importance of the three sets of predictor variables used for the construction of the ANNs is depicted in Figure 8 (models of students’ satisfaction with the online teaching method). The reduced Boruta set of input variables (Figure 8b) shows a negative contribution to the output variable, where the TV sounds’ interference with synchronous activities (SYNCH_TV; 32.16%) and the general interference with synchronous activities (INT_SYNC; 23.89%) are the factors that contribute the most. This negative contribution is coherent with the order of the possible responses of the Likert scales from low to high on noise interference. Therefore, high values of these variables may lead to low degrees of students’ satisfaction with online learning. The relative importance of the set of variables selected by the mRMR method is shown in Figure 8c. The most important variable of that model is related to noise’s making the students lose track of the lesson (SYNC THR; 49.28%). The second variable that contributes the most has a positive contribution, which is in line with the type of answers allowed; the more the students endure noise, the higher their satisfaction with online learning is (ENDURE_NOI; 17.57%). As happens with linear models, the relative importance of the ANN built with the input variables selected by the Boruta method (Figure 8a) is more difficult to interpret, because of the high number of input variables used.

#### 3.4.2. ANN Model of the Noise Interference with Autonomous Activities

Similar to the procedure followed to calculate the ANNs to predict students’ satisfaction with the online learning method, a reduced Boruta set of variables was chosen in order to compare ANN models calculated with the same number of predictors. The three sets of input variables are listed in descending order of importance, in Table 4. Table 5 shows the correlation coefficients and the RMSE of the best-performing ANN models to predict noise interference with autonomous activities.

The ANN model calculated using Boruta-selected variables as predictors has a slightly stronger mean correlation coefficient (r_Boruta_ = 0.8393) than the others (r_ReducedBoruta_ = 0.8187 and r_mRMR_ = 0.8222) (Table 5) because of the large number of predictors used for building the model. No remarkable differences can be found in the correlation coefficients of the ANN models using the mRMR or using the set of variables of reduced Boruta. For the three models, the RMSE is similar for the train, validation, and test sets of data.

**Figure 9:**
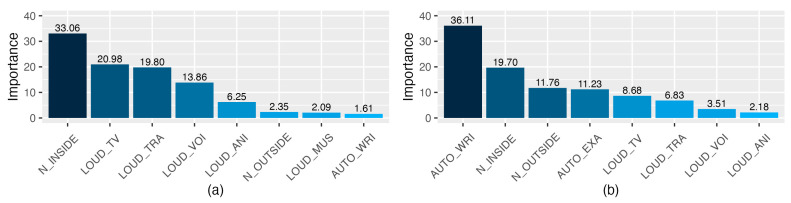
Percentage of the relative importance of the predictors of the ANNs on the noise interference with the autonomous activities calculated with the Olden et al. method [61], using as predictors the variables selected by mRMR (**b**) and the set of reduced Boruta variables (**a**). Values higher than zero mean a positive relative contribution, and those lower than zero mean a negative relative contribution.

The relative importance of two sets of predictor variables used for the construction of the ANNs is depicted in Figure 9 (models of the noise interference with the autonomous activities). The reduced Boruta set of input variables (Figure 9a) shows a positive contribution to the model output, where the general interference of noise coming from inside the house (N_INSIDE; 33.06%) and the perceived loudness of the TV/radio/household appliances (LOUD_TV; 20.98%) are the factors that contribute the most. All the variables contribute positively to the output, which is in coherence with the possible responses and their order on the Likert scale (e.g., the higher the perceived loudness of the TV, the higher the degree of interference with engaging in synchronous activities).

The relative importance of the set of variables selected by the mRMR method is shown in Figure 9b. The most important variable of that model is related to the interference of noise when engaging in writing activities (AUTO_WRI; 36.11%), followed by interferences from the inside (N_INSIDE; 19.70%) and outside noise (N_OUTSIDE; 11.76%). Remarkably, the last variable in order of importance of the ANN built with the reduced Boruta is the first one in the model built with the variables selected by the mRMR method. As happens with linear models, the relative importance of the ANN built with the input variables selected by the Boruta method is more difficult to interpret because of the high number of input variables used, and consequently, it was not depicted in Figure 9.

#### 3.4.3. ANN Model of the Noise Interference with Synchronous Activities

Table 6 shows the input variables in descending order of importance of the best-performing models of ANN calculated for predicting the noise interference with synchronous activities. Table 7 shows the correlation coefficients and the RMSE for the train, test, and validation sets of the best-performing models to predict the noise interference with the synchronous activities.

The ANN calculated using Boruta-selected variables as predictors has a mean correlation coefficient (r_Boruta_ = 0.8568) higher than the other models (Table 7), but also the number of predictors is larger: 27 variables of the ANN–Boruta combination (from a total of 30), in comparison with 6 variables used for the other models (r_ReducedBoruta_ = 0.8282 and r_mRMR_ = 0.8385). As for the previous ANN models, no remarkable differences can be found in the RMSE and the correlation coefficient of the ANN models using mRMR or the reduced Boruta set of variables.

**Figure 10:**
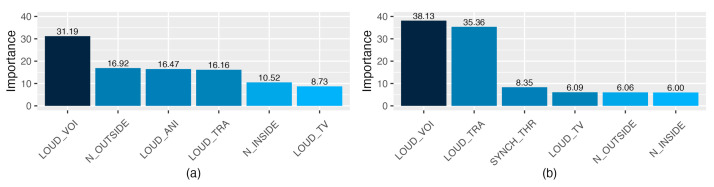
Percentage of the relative importance of the predictors of the ANNs on the noise interference with the synchronous activities calculated with the Olden et al. method [61], using as predictors the variables selected by mRMR (**b**) and the set of reduced Boruta variables (**a**). Values higher than zero mean a positive relative contribution, and those lower than zero mean a negative relative contribution.

Figure 10 shows the relative importance of two sets of predictor variables used for the construction of the ANNs for the models on the interference of noise with synchronous activities. Since the interpretation of the contribution of the input variables selected by Boruta is not so straightforward, because of a large number of predictors, the relative importance of the ANN input variables was not depicted in Figure 10.

The reduced Boruta set of input variables (Figure 10a) shows a positive contribution to the model output: the perceived loudness of the voices was the predictor with the highest relative importance (LOUD_VOI; 31.19%), followed by the interference with academic activities caused by the outside noise (N_OUTSIDE; 16.92%). As for the model of noise interference with autonomous activities, the positive contribution to the output is in line with the possible responses and their order in the Likert scale. The relative importance of the set of variables selected by the mRMR method is shown in Figure 10b. The variables with larger relative importance are related to the perceived loudness of voices and traffic (LOUD_VOI; 38.13% and LOUD_TRA; 35.36%). The degrees of interference of the noise outside and inside the home have the lowest percentages in the construction of the model (N_OUTSIDE; 6.06% and N_OUTSIDE; 6.00%).

## 4. Discussion

This paper evaluates the effect of the acoustic environment on students’ satisfaction with the online learning method, using the data provided by the students through an online survey. The capability of the acoustic environmental factors to predict the students’ satisfaction was evaluated by calculating models of ANN. To avoid redundant or irrelevant information and to optimize the computational timing, the input variables of the ANNs were chosen by two feature-selection methods, Boruta and mRMR. Furthermore, two ANN models were calculated to evaluate factors related to the acoustic environment, in particular the noise interference with autonomous and synchronous activities, using also only acoustic variables as predictors. These models allow us to compare the results of predicting acoustic-related and non-acoustic-related variables only with factors regarding the home soundscape.

### 4.1. Descriptive Statistics

Before calculating the predictive models, descriptive statistics were performed. In our study, students belonging to the initial and final semesters tend to be more satisfied with the online teaching method than the others. This is in accordance with the results of a recent longitudinal study on student satisfaction that was conducted in Germany in the two semesters of COVID-19 lockdown [70]. The study showed that younger students tend to feel more satisfied with the study conditions. The authors argue that because they are enrolled in the lower semesters, they may feel less frustrated, as they have less experience with the university study conditions and are therefore more satisfied. Strong motivations (such as when the student has to finish their degree) also lead to higher student satisfaction [71], probably because the enthusiastic students may see changes from the pandemic conditions in a positive way [70].

The interference of different sound sources (‘Animals’, ‘Music’, ‘Traffic’, ‘TV/Radio/Household Appliances’, and ‘Voices’) on the academic activities was analysed according to the student’s appraisals. Results show that more that 50% of participants consider that music does not interfere at all in their academic activities. This is related to the outcomes of different research on listening to music as background noise. For example, in a recent study conducted by Krause et al., many participants reported listening to music for emotions/problems and avoidance/disengagement [72], as music produces a positive mood change and enhances the perception of the work developed [73]. The same research group carried out a study using data collected in the periods before and during the COVID-19 lockdown, which showed that listening to music was positively associated with life satisfaction [74]. It also improves efficiency and productivity when it is reproduced while conducting repetitive work [75]. When referring to academic implications, research studies suggest that music improves cognitive and task performance. For example, music enhances the results of intelligence test [76], has a neutral [77] or positive influence on reading comprehension [78], and improves the results of a task on a lecture with classical music as background noise [79]. These results depend, however, on the type of music played and the preferences [80] and implications of the listeners [81]. All this research led to the conclusion that (because normally the students themselves decide when and what kind of music to listen to) music can be seen as a “wanted sound” [82], and it is hardly considered a background disturbance.

According to the distribution of the student’s appraisals, it may seem that noise does not excessively affect their engagement in academic activities. However, it is necessary to assess these data from another perspective; for instance, research dealing with environmental noise has led to the conclusion that people who report being highly annoyed by traffic noise may present health-related problems [83,84]. In this regard, the International Committee for the Biological Effects of Noise (ICBEN) team, Community Response to Noise [85,86], established that scores on traffic noise annoyance ≥ 4 on a 5-point Likert scale may be considered problematic, which has led to numerous studies on the topic [87]. If we follow a similar criterion and evaluate our results according to the students who report that noise interferes highly in their academic activities (scores ≥ 4), we can see that voice noises highly interfere with engaging in synchronous activities for 29% of students, and animal noises do so for 21.4% (Table 8). During the period in which the surveys were conducted, there were restrictions on the circulation of vehicles, so possibly in different periods, interference from traffic noise will be larger [88], especially given that outdoor noise levels in Quito with normal traffic conditions are very high [89]. Subsequently, there is a percentage of students highly affected by different noise sources that we should not underestimate.

### 4.2. Feature-Selection Methods

Upon an analysis of the feature-selection methods used, although the Boruta method offers better results in the ANN, it selects an excessive number of variables and entails a longer calculation time compared with the mRMR method, whose results are immediate (e.g., for the satisfaction model, t_mRMR_ = 0.000 s and t_Boruta_ = 120.325 s, calculated with an Intel Core i7-9750H processor, CPU 2.60 GHz, 6 main processors, 12 virtual processors). Furthermore, the mRMR method in R shows us the inferred network topology, which facilitates the evaluation of the existing relationships between the selected variables in a very intuitive way (a great advantage when interpreting our model).

Features selected for the student‘s satisfaction model: variables related to noise interfering with comprehensive reading (AUTO_COM) and taking exams (AUTO_EXA; Score_Satisfaction = 0.0507) and the frequency of noises that make them lose attention (SYNCH_ATT; Score_Satisfaction = 0.0514) or lose track of the classes (SYNCH_THR; Score_Satisfaction = 0.0600) are factors selected by both the Boruta and mRMR methods, so their importance in students’ satisfaction is remarkable.

Features selected for the models of the noise interference with autonomous and synchronous activities:

(1) Noises coming from the interior (N_INSIDE; Score_Auto = 0.3398, Score_Synch = 0.3906) and the exterior (N_OUTSIDE; Score_Auto = 0.1791, Score_Synch = 0.1685) of the houses are factors that play fundamental roles in noise interference prediction (in synchronous and autonomous activities), as they were selected by both algorithms. The relevance of outside noise may be caused by the poor acoustic insulation of the houses, an aspect pointed out in a previous study conducted by our research group [89]. This study highlights that the acoustic insulation of traditional constructive systems of Quito can be around 6 dB less than the Spanish ones, which may explain that the N_OUTSIDE factor was selected by all the feature-selection methods and for all the predictive models calculated.

(2) According to the variables selected for the model autonomous academic activities, it seems that the tasks that are most affected by noise are writing reports or essays (AUTO_WRI; Score_Auto = 0.0295) and taking exams (AUTO_EXA; Score_Auto = 0.0518). For the synchronous activities, however, the frequency of losing track of the class (SYNCH_THR; Score_Synch = 0.1956), although selected only by the mRMR method, plays an important role in the conformation of the model and also affects the degree of satisfaction with the online learning modality.

(3) The variables on the perceived loudness of the noise sources (e.g., LOUD_VOI, LOUD_TRA, etc.) are more relevant than the ones related to the specific interference of the noise sources (AUTO_VOI, AUTO_TRA, SYNCH_ANI, etc.), since the loudness of each noise source was selected at least by one feature-selection method (referring to red Boruta and mRMR), while no variable on the interference of the noise sources was selected.

### 4.3. Artificial Neural Network Models

**Figure 11:**
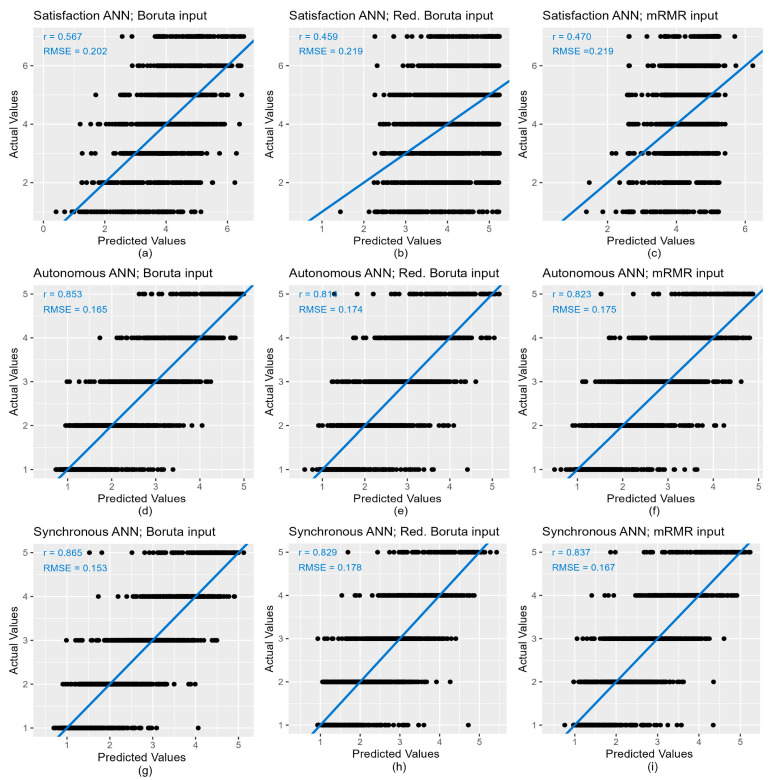
Predicted versus actual values of the ANNs under study, according to the training data set. The title of each subgraph indicates the predicted variable (satisfaction with the online learning—**first row** (**a**–**c**); noise interference with autonomous activities—**second row** (**d**–**f**); and noise interference with synchronous activities—**third row** (**g**–**i**)), and the input features selected (with Boruta method—**first column** (**a**,**d**,**g**); with reduced Boruta—**second column** (**b**,**e**,**f**); and with mRMR method—**third column** (**c**,**f**,**i**)). The blue line is the estimated regression line. The correlation coefficients (r) and root mean square errors (RMSEs) are shown in the top-left corner of each subgraph.

Figure 11 shows predicted versus actual values of the ANNs under study for the training data set. When comparing ANN models built with the same number of variables (using red Boruta and mRMR as predictors), it can be appreciated that although the performance of the models is similar, the ones built with the predictors selected by mRMR were slightly better for the three models, according to the outcomes of the training set.

The apparently low correlation coefficient between the predicted and measured variables obtained for the ANN model of students’ satisfaction (r_Boruta_ = 0.567, r_ReducedBoruta_ = 0.459, and r_mRMR_ = 0.470 for the training data set) is related to the type of predictors used (Figure 11). The information provided by the input variables was only about the perceived acoustic environment, and therefore, other aspects that could affect students’ satisfaction (e.g., interaction with other students or with the teaching staff [90], quality of the e-contents [91], tools used for learning/teaching [92], etc.) were not considered. Another factor that influences the performance of the model is the type of variables used, as explanatory and target variables were ordinal. This fact makes more difficult obtaining a good fit in comparison with models built with numerical predictors.

The correlation coefficients are higher for the models of noise interference with academic activities (than for the models of students’ satisfaction), as both the predictors and the independent variables were related to the acoustic environment. However, these correlation coefficients are not as near 1 as expected, probably because there are other factors that may affect the noise perception, as pointed out in different studies related to noise perception (e.g., audiovisual interaction [93,94], listeners’ activity [95], dynamics of the places [96], etc.).

The relative importance of the variables was calculated using the Olden et al. method. Because neural networks are considered black boxes, this method sheds some light when interpreting specific models of neural networks and the role of the variables in their conformation. However, because each model initially uses random weights (which can be controlled by setting a seed), the interpretation of the results may not agree with the expected influence of the input variables on the independent variable (in direction and weight). This happens because the objective of the ANN is to obtain the most adjusted results possible, without taking into account the behaviour of the variables. Nevertheless, in our case (except for the relative importance of the models that used the Boruta method with all the selected variables), the relative importance of the models was coherent in the direction of the variables, with expected results.

For the models of students’ satisfaction, the noise that makes them lose track of the class (SYNCH_THR) is among the four most important variables in all models, which highlights the importance of this variable in students’ satisfaction. Regarding the noise interference with autonomous activities, the noise coming from inside the house (N_INSIDE) is one of the two variables most important when the input variables are calculated with the mRMR and red Boruta. For the model of the noise interference with synchronous activities, the perceived loudness of voices (LOUD_VOI) is the aspect with higher relative importance when using the input variables calculated with the previously mentioned methods, because voices probably interfere with listening to synchronous lessons.

**Figure 12:**
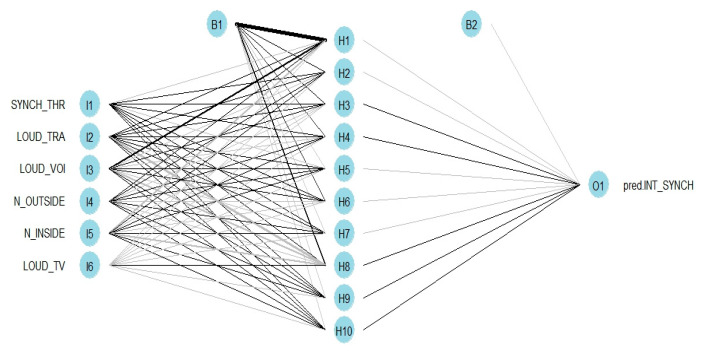
Artificial neural network representation of the model for the noise interference with synchronous activities. The black lines represent positive weights and the grey ones negative. Line thickness is the relative weight with respect to the other variables.

There are other methods that allow the interpretation of the network, although sometimes it is not easy to extract general conclusions from them. For example, Figure 12 shows a diagram of the ANN model for noise interference with synchronous activities, using as input data the set of variables selected by the mRMR method. The black lines represent positive weights and the grey ones negative. Line thickness is the relative weight with respect to the other variables. The hidden layer is labelled from H1 to H10, according to the number of neurons. However, even if we had included the value of the weights, it would have been difficult to interpret the role of each explanatory variable in all the models’ outcomes.

**Figure 13:**
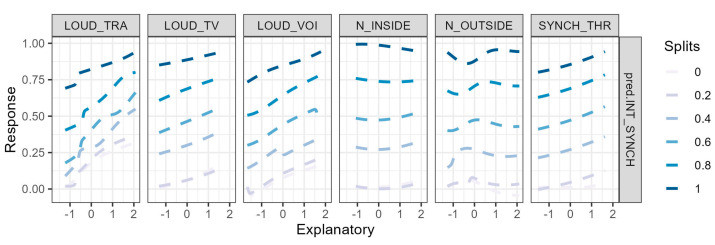
Profile method applied to the ANN model of the noise interference with synchronous activities, using as input variables the ones selected by the mRMR method. The predicted variable is split into quantiles.

The Lek profile method is another procedure to evaluate the outcomes according to the changes of the explanatory variables. It studies each input variable and the response when the other input variables are lock at certain fixed values [97]. The outcomes can be represented at a certain number of equal intervals. For example, Figure 13 represents the profile method applied to the previous model. The variables were kept constant at the quantiles of the target. However, the explanation of the graphic is not so straightforward, because the response variable, as it is represented, is not directly linked to the different categories of the explanatory variables. However, this method provides a lot of information on the link between the explanatory variables and the outcomes of the ANN. We can see that the higher the values of the variables LOUD_TRA, LOUD_TV, LOUD_VOI, and SYNCH_THR, the higher the values of the response variable, which is in line with the results of the relative importance of the variables. For N_INSIDE and N_OUTSIDE, however, the trend is not so clear, although there is a slight growth comparing the minimum and maximum values of the explanatory variables for some of the quantiles of the predicted variable.

### 4.4. Limitations of the Study

The current study presents some limitations that we highlight: (1) The sample of our study is based on university students from the Universidad de Las Américas, a private university, and was conducted during the COVID-19 lockdown, aspects that limit the study’s generalizability. (2) We used ordinal variables, expressed through numbers for the different categories. The numerals showed the order of the categories, but they do not necessary indicate equal intervals between them. For example, if the response items include ‘4′ = “neither satisfied nor unsatisfied,” ‘3′ = “Slightly unsatisfied,”, ‘2′ = “moderately unsatisfied”, or ‘1′ = “very unsatisfied”, we cannot assume that the increment in satisfaction from ‘1′ to ‘2′ is the same as the increment in satisfaction from ‘3′ to ‘4′ [98], and therefore, this can lead to misinterpretations of the data. (3) The objective of the study was to evaluate the influence of the study on students’ satisfaction with the online learning modality. However, this objective itself is a limitation of the study, which we knew from the beginning was going to condition the results, in that many other aspects that can influence the students’ satisfaction have not been considered.

## 5. Conclusions

Sound is the basic element thanks to which students can communicate both with the teaching staff and with their classmates, but it can also be distracting. Despite the crucial role that it plays in learning, only a few studies have been commissioned to evaluate the effects of the acoustic environment on online teaching. In this study, how sound affects the degree of student satisfaction with online education, together with the noise interferences on different tasks, was evaluated.

To predict the degree of students’ satisfaction with the online learning method, ANNs were used with different predictor variables obtained through feature-selection methods. These models resulted in correlation coefficients (of the train set) between 0.567 (RMSE = 0.202) for Boruta and 0.459 (RMSE = 0.219) for red Boruta, depending on the number of predictor variables (27 and 7, respectively). Although it may seem that these are low correlation values, we must take into account that only variables related to the perceived acoustic environment were considered and that all the variables were ordinal. Therefore, they are very promising results that lay the foundations for future research on the acoustic environment in which students are immersed and its relationship with student satisfaction (applying or improving the applied methods used in the present study). However, more research is needed in order to consider, for example, other aspects of the environment in which students engage in their tasks (e.g., comfort of the furniture, thermal or illumination conditions, etc.). In this case, using the Boruta model leads to a better result; however, the interpretation of the variables within the network is not straightforward.

Additionally, the potential of artificial neural network models to predict noise interference with autonomous and synchronous activities was also evaluated. These models showed better performance (which is logical given that the variables to be predicted were related to the acoustic environment). For the interference of noise in autonomous activities, the mean correlation coefficients of the models oscillated between 0.853 (RMSE = 0.165, 27 variables) and 0.814 (RMSE = 0.174, 8 variables), and for the models of synchronous activities, between 0.865 (RMSE = 0.153, 27 variables) and 0.829 (RMSE = 0.178, 6 variables). Therefore, we believe that neural networks have good potential for predicting the degree of noise interference with synchronous and autonomous activities and that feature-selection methods are of great help to promote better performance for prediction models. For these models, although the performance of the ANN calculated with Boruta was slightly higher, we consider that the network built with the input variables selected by the mRMR method led to a more efficient model, with less risk of overfitting.

According to the results, those considered relevant by both feature-selection models for the model of students’ satisfaction were the noises interfering with comprehensive reading and taking exams and the frequency of noises that make students lose the attention or lose track of the classes.

The loudness of the TV/radio/home appliances, traffic, and voices were the other important predictors of noise interference on autonomous and asynchronous activities, together with the general noises coming from inside and outside of homes.

The combination of Boruta and mRMR feature-selection methods has led to interesting results, especially in the selection of common variables and, after building the ANNs models, in the relative importance of the variables. However, given the different aspects of the performance of the model (calculation time, risk of overfitting, etc.), the models built with the features selected by the mRMR are more efficient and easier to interpret.

This study’s results represent the first step for the development of a methodology that allows for studying the effects of the acoustic environment on the degree of the students’ satisfaction with the online learning methods. As sounds may act as distractors of the student’s attention, working to obtain a favourable acoustic environment (e.g., without unexpected acoustic events, excessive reverberation, or background noise and without audio problems owing to internet speed or network quality) may guarantee the correct transmission of the message from the sender to the receiver and increase students’ satisfaction with the online learning modality. Consequently, it should be advisable to promote awareness campaigns on noise problems and inform people of measures that students and their families could take to reduce these levels inside homes.

## Figures and Tables

**Table 1:** Selected features with the mRMR method for the models of students’ satisfaction with online learning (Satisfaction; SAT_ONLINE) and the models for the interference of noise with autonomous (Auto; INT_AUTO) and synchronous (Synch; INT_SYNCH) academic activities. The score of each variable is also shown. The meanings of the acronyms of the selected variables are shown in Table A1.

Satisfaction	Score	Auto	Score	Synch	Score
SYNCH_THR	0.0600	N_INSIDE	0.3398	N_INSIDE	0.3906
SYNCH_ATT	0.0514	LOUD_VOI	0.2408	LOUD_VOI	0.2644
AUTO_COM	0.0513	LOUD_TV	0.1885	SYNCH_THR	0.1956
AUTO_EXA	0.0507	N_OUTSIDE	0.1791	LOUD_TV	0.1836
ORDER_IN_2	0.0010	AUTO_EXA	0.0518	N_OUTSIDE	0.1685
INTER_AUDIO	−0.0001	LOUD_TRA	0.0443	LOUD_TRA	0.0436
ENDURE_NOI	−0.0007	AUTO_WRI	0.0295		
		LOUD_ANI	0.0289		

**Table 2:** Acronyms of the variables selected for the model of students’ satisfaction with the online learning method using the Boruta (first column) and mRMR (third column) feature-selection methods listed by descending order of importance (using the Boruta relative importance and the mRMR scores, respectively). The second column shows a list with the same number of variables selected by the mRMR method, but it is composed of the first Boruta-selected variables in descending order of relative importance. The meanings of the acronyms of the variables selected for the models are shown in Table A1.

Satisfaction Model		
Boruta	Reduced Boruta	mRMR
SYNCH_THR, AUTO_EXA, SYNCH_ATT, AUTO_WRI, INT_SYNC, SYNCH_TV, AUTO_COM, AUTO_WOR, INT_AUTO, AUTO_GRA, SYNCH_ANI, AUTO_ANI, LOUD_VOI, SYNCH_VOI, SYNCH_ANS, AUTO_MAT, AUTO_VOI, SYNCH_NOT, AUTO_TV, SYNCH_MUS, LOUD_TV, AUTO_MUS, LOUD_MUS, ENDURE_NOI, N_OUTSIDE, SYNCH_TRA, LOUD_ANI	SYNCH_THR, AUTO_EXA, SYNCH_ATT, AUTO_WRI, INT_SYNC, SYNCH_TV, AUTO_COM	SYNCH_THR, SYNCH_ATT, AUTO_COM, AUTO_EXA, ORDER_IN_2, INT_AUDIO, ENDURE_NOI

**Table 3:** ANN results for the best-performing models (among 100) for students’ satisfaction with the online teaching method, using the variables selected by Boruta and mRMR, and the set of reduced Boruta. The table shows the correlation coefficient and the root mean square error (RMSE) for the train, test, and validation sets.

ANN Model of Students’ Satisfaction with the Online Teaching Method							
	Train		Validation		Test		Mean
	Corr. Coef.	RMSE	Corr. Coef.	RMSE	Corr. Coef.	RMSE	Corr. Coef.
Boruta + ANN	0.5667	0.2019	0.5138	0.2089	0.4834	0.2147	0.5213
Reduced Boruta + ANN	0.4585	0.2192	0.4941	0.2218	0.4169	0.4029	0.4565
mRMR + ANN	0.4698	0.2188	0.4828	0.2268	0.4151	0.3943	0.4559

**Table 4:** Acronyms of the variables selected for the model of the noise interference with the autonomous activities using the Boruta (first column) and mRMR (third column) feature-selection methods listed by descending order of importance (using the Boruta relative importance and the mRMR scores, respectively). The second column shows a list with the same number of variables selected by the mRMR method, but it is composed of the first Boruta-selected variables. The meanings of the acronyms of the variables selected for the models are shown in Table A1.

Boruta	Reduced Boruta	mRMR
N_INSIDE, N_OUTSIDE, LOUD_VOI, LOUD_TV, LOUD_ANI, LOUD_TRA, LOUD_MUS, AUTO_WRI, AUTO_EXA, AUTO_WOR, SYNCH_THR, SYNCH_ATT, AUTO_VOI, AUTO_COM, AUTO_TV, AUTO_GRA, AUTO_MAT, SYNCH_NOT, SYNCH_ANS, SYNCH_TV, SYNCH_VOI, AUTO_MUS, AUTO_ANI, AUTO_TRA, SYNCH_ANI, SYNCH_MUS, SYNCH_TRA, INTER_AUDIO	N_INSIDE, N_OUTSIDE, LOUD_VOI, LOUD_TV, LOUD_ANI, LOUD_TRA, LOUD_MUS	N_INSIDE, LOUD_VOI, LOUD_TV, N_OUTSIDE, AUTO_EXA, LOUD_TRA, AUTO_WRI

**Table 5:** ANN results for the best-performing models of noise interference with autonomous activities using the variables selected by Boruta, mRMR, and the set of reduced Boruta. The table shows the correlation coefficient and the root mean square error (RMSE) for the train, validation, and test sets.

ANN Model of the Noise Interference while Performing Autonomous Academic Activities							
	Train		Validation		Test		Mean
	Corr. Coef.	RMSE	Corr. Coef.	RMSE	Corr. Coef.	RMSE	Corr. Coef.
Boruta + ANN	0.8526	0.1649	0.8562	0.1572	0.8090	0.1766	0.8393
Reduced Boruta + ANN	0.8142	0.1742	0.8479	0.1602	0.7940	0.1823	0.8187
mRMR + ANN	0.8225	0.1753	0.8534	0.1639	0.7908	0.1824	0.8222

**Table 6:** Acronyms of the variables selected for the model of the noise interference with the synchronous activities using the Boruta (first column) and mRMR (third column) feature-selection methods listed by descending order of importance (using the Boruta relative importance and the mRMR scores, respectively). The second column shows a list with the same number of variables selected by the MRMR method, but it is composed of the first Boruta-selected variables. The meanings of the acronyms of the variables selected for the models are shown in Table A1.

Boruta	Reduced Boruta	mRMR
N_INSIDE, N_OUTSIDE, LOUD_VOI, LOUD_TV, LOUD_ANI, LOUD_TRA, SYNCH_THR, SYNCH_ATT, LOUD_MUS, AUTO_EXA, SYNCH_NOT, AUTO_WOR, AUTO_WRI, SYNCH_VOI, AUTO_COM, SYNCH_ANS, AUTO_MAT, AUTO_TV, SYNCH_TV, SYNCH_TRA, AUTO_GRA, AUTO_VOI, SYNCH_ANI, AUTO_TRA, AUTO_ANI, AUTO_MUS, SYNCH_MUS	N_INSIDE, N_OUTSIDE, LOUD_VOI, LOUD_TV, LOUD_ANI, LOUD_TRA	N_INSIDE, LOUD_VOI, SYNCH_THR, LOUD_TV, N_OUTSIDE, LOUD_TRA

**Table 7:** ANN results for the best-performing models of noise interference with synchronous activities using the variables selected by Boruta, mRMR, and the set of reduced Boruta. The table shows the correlation coefficient and the root mean square error (RMSE) for the train, validation, and test sets.

ANN Model of the Noise Interference While Performing Synchronous Academic Activities							
	Train		Validation		Test		Mean
	Corr. Coef.	RMSE	Corr. Coef.	RMSE	Corr. Coef.	RMSE	Corr. Coef.
Boruta + ANN	0.8647	0.1532	0.8790	0.1532	0.8266	0.1685	0.8568
Reduced Boruta + ANN	0.8290	0.1778	0.8543	0.1620	0.8013	0.1800	0.8282
mRMR + ANN	0.8369	0.1670	0.8655	0.1683	0.8131	0.1751	0.8385

**Table 8:** Percentage of students who report that noise highly interferes with their engaging in autonomous and synchronous activities (scores of ≥4).

Synchronous		Autonomous	
Scores > 3		Scores > 3	
SYNCH_TRA	15.54%	AUTO_TRA	14.86%
SYNCH_VOI	28.99%	AUTO_VOI	24.63%
SYNCH_TV	16.11%	AUTO_TV	15.70%
SYNCH_MUS	11.99%	AUTO_MUS	12.19%
SYNCH_ANI	21.44%	AUTO_ANI	19.58%

## Data Availability

Not applicable.

## Appendix A

**Table A1:** Questions on the students’ acoustic environment at home and the students’ satisfaction with the online learning modality used for the present study.

Questions	Measurement Scale
In the last month, how satisfied have you felt with the online learning method? (SAT_ONLINE)	Very Unsatisfied (1), Very Satisfied (7)
2. In the last month, how loud have the following types of sounds been during your academic activities (in general)? Animals (LOUD_ANI) Music (LOUD_MUS) Traffic (LOUD_TRA) TV/Radio/Household appliances (LOUD_TV) Voices (LOUD_VOI)	I did not hear them (0), Very low (1), Neither high nor low (3), Very high (5)
3. In the last month, how much have the following types of sounds interfered with synchronous lessons? Animals (SYNCH_ANI) Music (SYNCH_MUS) Traffic (SYNCH_TRA) TV/Radio/Household appliances (SYNCH_TV) Voices (SYNCH_VOI)	I did not hear them (0), They did not interfere (1), Extremely (5)
4. In the last month, how much have the following types of sounds interfered with your autonomous tasks? Animals (AUTO_ANI) Music (AUTO_MUS) Traffic (AUTO_TRA) TV/Radio/Household appliances (AUTO_TV) Voices (AUTO_VOI)	I did not hear them (0), They did not interfere (1), Extremely (5)
5. In this last month, how often did the sounds of your home environment during synchronous lessons ...? Make you lose your attention? (SYNCH_ATT) Cause you to not hear clearly? (SYNCH_NOT) Make you lose the thread of the class? (SYNCH_THR) Prevent you from answering the questions in time? (SYNCH_ANS)	Never (1), Very often (5)
6. In the last month, how much have the sounds of your domestic environment interfered with the following autonomous tasks? Problem-solving (AUTO_MAT) Working in groups (AUTO_WOR) Comprehensive reading (AUTO_COM) Writing (AUTO_WRI) Making graphs/diagrams/drawings/models (AUTO_GRA) Exam (practical-theoretical) (AUTO_EXA) *	They did not interfere (1), Extremely (5)
7. In the past month, to what extent has the noise bothered you in your academic activities? Coming from inside your home? (N_INSIDE) Coming from outside your home? (N_OUTSIDE)	They did not interfere (1), Extremely (5)
8. In the last month, how much have the sounds of your domestic environment interfered? In your synchronous lessons? (INT_SYNCH) In your autonomous tasks? (INT_AUTO)	They did not interfere (1), Extremely (5)
9. In the past month, how often have you experienced noise connection problems attributable to your internet provider? (INTER_AUDIO)	Never (1), Very often (5)
10. How long would I be able to endure a noisy environment? (ENDURE_NOI)	Just a little (1), A lot (5)
11. Order the following factors according to the degree of interference with their academic performance in the last month, the first being the one that has interfered the most. Order of each variable: Comfort of your home environment (ORDER_IN_1) Acoustic environment (ORDER_IN_2) Thermal environment (ORDER_IN_3) Internet service quality (ORDER_IN_4) Stress (ORDER_IN_5)	

* Taking exams was classified as an autonomous activity because although there may be interaction with the teacher, they did not actively participate in the development of the exams.

## References

[1] Honeyman M., Miller G. (1993). Agriculture Distance Education: A Valid Alternative for Higher Education?. Proceedings of the 20th Annual National Agricultural Education Research Meeting.

[2] Holmberg B. (1995). The Evolution of the Character and Practice of Distance Education. Open Learn. J. Open Distance E-Learn..

[3] Pacheco C. Historia de TIC y E-Learning. https://www.timetoast.com/timelines/historia-de-las-tic-en-la-educacion-y-del-e-learning-0a489cfc-91a7-41e9-9809-c38565863d2e.

[4] Osial M., Pregowska A., Masztalerz K., Garli M. (2021). A Worldwide Journey Through Distance Education—From the Post Office to Virtual, Augmented and Mixed Realities, and Education during the COVID-19 Pandemic. Educ. Sci..

[5] Bušelić M. (2012). Distance Learning–Concepts and Contributions. Oeconomica Jadertina.

[6] University of Baltimore Distance Education Timeline. https://blogs.ubalt.edu/academicinnovation/wp-content/uploads/sites/38/2013/12/Distance-Education-Timeline.pdf.

[7] Montecinos M.V., Ganga-contreras F.A. (2020). Educación a Distancia En Latinoamérica: Algunos Antecedentes Históricos de Su Desarrollo. Espacios.

[8] Rumble G. (1985). Distance Education in Latin America: Models for the 80s. Distance Educ..

[9] (2014). Acoustics–Soundscape.

[10] WHO Regional Office for Europe (1999). Guidelines for Community Noise.

[11] Dockrell J.E., Shield B.M. (2006). Acoustical Barriers in Classrooms: The Impact of Noise on Performance in the Classroom. Br. Educ. Res. J..

[12] Gremp M.A., Easterbrooks S.R. (2018). A Descriptive Analysis of Noise in Classrooms across the U.S. and Canada for Children Who Are Deaf and Hard of Hearing. Volta Rev..

[13] Braat-eggen E., Reinten J., Hornikx M., Kohlrausch A. (2021). The Effect of Background Noise on a “Studying for an Exam” Task in an Open-Plan Study Environment: A Laboratory Study. Front. Built Environ..

[14] Hétu R., Truchon-Gagnon C., Bilodeau S.A. (1990). Problems of Noise in School Settings: A Review of Literature and the Results of an Exploratory Study. J. Speech-Lang. Pathol. Audiol..

[15] Shield B., Dockrell J.E. (2004). External and Internal Noise Surveys of London Primary Schools. J. Acoust. Soc. Am..

[16] Cohen S., Krantz D.S., Stokols D. (1980). Physiological, Motivational and Cognitive Effects of Aircraft Noise on Children: Moving from the Laboratory to the Field. Am. Psychol..

[17] Stansfeld S., Haines M., Brown B. (2000). Noise and Health in the Urban Environment. Rev. Environ. Health.

[18] Bullinger M., Hygge S., Evans G.W., Meis M., Mackensen S. (1999). The Psychological Cost of Aircraft Noise for Children for Children. Zent. Hyg. Umweltmed..

[19] Money L.E., Ramkissoon I. (2020). Effects of Secondhand Smoke Exposure and Noise Exposure on Tinnitus Occurrence in College Students and Adolescents. J. Am. Acad. Audiol..

[20] Brännström K.J., Lyberg-Åhlander V., Sahlén B. (2022). Perceived listening effort in children with hearing loss: Listening to a dysphonic voice in quiet and in noise. Logop. Phoniatr. Vocology.

[21] Shield B.M., Dockrell J.E. (2008). The Effects of Environmental and Classroom Noise on the Academic Attainments of Primary School Children. J. Acoust. Soc. Am..

[22] Mukhtar K., Javed K., Arooj M., Sethi A. (2020). Advantages, Limitations and Recommendations for Online Learning during COVID-19 Pandemic Era. Pak. J. Med. Sci..

[23] Pham L., Limbu Y.B., Bui T.K., Nguyen H.T., Pham H.T. (2019). Does E-Learning Service Quality Influence e-Learning Student Satisfaction and Loyalty ? Evidence from Vietnam. Int. J. Educ. Technol. High. Educ..

[24] Alqurashi E. (2019). Predicting Student Satisfaction and Perceived Learning within Online Learning Environments. Distance Educ..

[25] Ghaderizefreh S., Hoover M.L. (2018). Student Satisfaction with Online Learning in a Blended Course. Int. J. Digit. Soc..

[26] Simpson J.M. (2012). Student Perceptions of Quality and Satisfaction in Online Education.

[27] Choy S., McNickle C., Clayton B. (2002). Learner expectations and experiences: An examination of student views of support in online learning. Q. Rev. Distance Educ..

[28] Rosenfeld G. (2005). A Comparison of the Outcomes of Distance Learning Students versus Traditional Classroom Students in the Community College. Ph.D. Thesis.

[29] Öztürk G., Karamete A., Çetin G. (2022). The Relationship between Pre-Service Teachers’ Cognitive Flexibility Levels The Relationship Between Pre-Service Teachers’ Cognitive Flexibility Levels and Techno-Pedagogical Education Competencies. Int. J. Contemp. Educ. Res..

[30] Wei H.-C., Chou C. (2020). Online Learning Performance and Satisfaction: Do Perceptions and Readiness Matter?. Distance Educ..

[31] Tri P.M., Uyen L.T.T., Uyen M.T.H., Trang T.T.T., Thuy N.T.C. (2022). EFL Students’ Challenges of Online Courses at Van Lang University during the COVID-19 Pandemic. Int. J. TESOL Educ..

[32] Borup J., Evmenova A.S. (2019). The Effectiveness of Professional Development in Overcoming Obstacles to Effective Online Instruction in a College of Education. Online Learn. J..

[33] Wang C. (2022). Comprehensively Summarizing What Distracts Students from Online Learning: A Literature Review. Hum. Behav. Emerg. Technol..

[34] Blasiman R.N., Larabee D., Fabry D. (2018). Distracted Students: A Comparison of Multiple Types of Distractions on Learning in Online Lectures. Scholarsh. Teach. Learn. Psychol..

[35] Ye J., Lee Y., He Z., Lee Y. (2022). The Relationship Among Expectancy Belief, Course Satisfaction, Learning Effectiveness, and Continuance Intention in Online Courses of Vocational-Technical Teachers College Students. Front. Psychol..

[36] Al-allaf O. (2015). Removing Noise from Speech Signals Using Different Approaches of Artificial Neural Networks. Int. J. Inf. Technol. Comput. Sci..

[37] Puyana Romero V., Ciaburro G., Maffei L. The Soundscape and the Degree of Match of a Waterfront with the Expectations Placed on It. The Cases Study of Naples and Brighton. Processing of the INTER-NOISE and NOISE-CON Congress and Conference Proceedings.

[38] Brocolini L., Waks L., Lavandier C., Marquis-Favre C., Quoy M., Lavandier M. Comparison between Multiple Linear Regressions and Artificial Neural Networks to Predict Urban Sound Quality. Proceedings of the 20th International Congress on Acoustics.

[39] Genaro N., Torija A., Ramos-Ridao A., Requena I., Ruiz D.P., Zamorano M. (2010). A Neural Network Based Model for Urban Noise Prediction. J. Acoust. Soc. Am..

[40] Mitchell A., Oberman T., Aletta F., Kachlicka M., Lionello M. (2021). Investigating Urban Soundscapes of the COVID-19 Lockdown: A Predictive Soundscape Modeling Approach. J. Acoust. Soc. Am..

[41] Aydoğdu Ş. (2020). Predicting Student Final Performance Using Artificial Neural Networks in Online Learning Environments. Educ. Inf. Technol..

[42] Kamal D., Alnagar F. (2020). Using Artificial Neural Network to Predicted Student Satisfaction in E-Learning. Am. J. Appl. Math. Stat..

[43] Osl M., Dreiseitl S., Cerqueira F., Netzer M., Pfeifer B., Baumgartner C. (2009). Demoting Redundant Features to Improve the Discriminatory Ability in Cancer Data. J. Biomed. Inform..

[44] Pudjihartono N., Fadason T., Kempa-liehr A.W. (2022). A Review of Feature Selection Methods for Machine Learning-Based Disease Risk Prediction. Front. Bioinform..

[45] Remeseiro B., Bolon-Canedo V. (2019). A Review of Feature Selection Methods in Medical Applications. Comput. Biol. Med..

[46] Amato F., Guignard F., Kanevski M. (2019). Feature Selection Using Simple and Efficient Machine Learning Models. Case Studies and Software Tools. Geophys. Res. Abstr..

[47] Puyana-Romero V., Maffei L., Brambilla G., Ciaburro G. (2016). Modelling the Soundscape Quality of Urban Waterfronts by Artificial Neural Networks. Appl. Acoust..

[48] Forman G. (2003). An Extensive Empirical Study of Feature Selection Metrics for Text Classification. J. Mach. Learn. Res..

[49] Bolón-Canedo V., Remeseiro B. (2020). Feature Selection in Image Analysis: A Survey. Artif. Intell. Rev..

[50] Siddiqi M.A., Pak W. (2020). Optimizing Filter-Based Feature Selection Method Flow for Intrusion Detection System. Electronics.

[51] Chandrashekar G., Sahin F. (2014). A survey on feature selection methods. Comput. Electr. Eng..

[52] Jay N.D., Papillon-cavanagh S., Olsen C., Bontempi G., Haibe-Kains B. (2021). MRMRe: An R Package for Parallelized MRMR Ensemble Feature Selection. R J..

[53] Gholami H., Mohammadifar A., Golzari S., Kaskaoutis D.G., Collins A.L. (2021). Collins, A. Using the Boruta Algorithm and Deep Learning Models for Mapping Land Susceptibility to Atmospheric Dust Emissions in Iran. Aeolian Res..

[54] Guerrero J.A. (2016). El Problema de La Dimensionalidad. Tema Portada.

[55] Kursa M.B., Rudnicki W.R. (2010). Feature Selection with the Boruta Package. J. Stat. Softw..

[56] Kursa M.B., Rudnicki W.R. (2022). Package “Boruta”: Algorithm for All Relevant Feature Selection. R J..

[57] Bordoloi M., Purkayastha B. (2016). Review on Feature Selection and Classification Using Neuro- Fuzzy Approaches. Int. J. Appl. Evol. Comput..

[58] Hagan M.T., Demuth H.B., Beale M.H., Demuth H. (2002). Neural Network Design.

[59] Ciaburro G., Venkateswaran B. (2017). Neural Networks with R: Smart Models Using CNN, RNN, Deep Learning, and Artificial Intelligence Principles.

[60] Fritsch S., Guenther F., Wright M.N., Suling M., Mueller S.M. (2022). Package ‘Neuralnet’: Training of Neural Networks. R J..

[61] Olden J.D., Jackson D.A. (2002). Illuminating the “Black Box”: A Ramdomization Approach for Understanding Variable Contributions in Artificial Neuronal Networks. Ecol. Modell..

[62] Nikentari N., Kurniawan H., Ahsan M., Setiyaningsih W. (2017). Analysis Resilient Algorithm on Artificial Neural Network Backpropagation. IOP Conf. Ser. J. Phys. Conf. Ser..

[63] Sritsch S., Guenther F. (2012). Training of Neural Networks. R J..

[64] Hornik K., Tinchcombe M., White H. (1989). Multilayer Feedforward Networks Are Universal Approximators. Neural Netw..

[65] Yu H., Samuels D.C., Zhao Y., Guo Y. (2019). Architectures and Accuracy of Artificial Neural Network for Disease Classification from Omics Data. BMC Genom..

[66] Bashiri M., Farshbaf Geranmayeh a. (2011). Tuning the Parameters of an Artificial Neural Network Using Central Composite Design and Genetic Algorithm. Sci. Iran..

[67] Fang X.L., Papaioannou N., Leach F., Davy M.H. (2021). On the Application of Artificial Neural Networks for the Prediction of NO x Emissions from a High-Speed Direct Injection Diesel Engine. Int. J. Engine Res..

[68] Olden J. (2004). An Accurate Comparison of Methods for Quantifying Variable Importance in Artificial Neural Networks Using Simulated Data. Ecol. Model..

[69] Puyana-Romero V., Maffei L., Brambilla G., Ciaburro G. (2016). Acoustic, Visual and Spatial Indicators for the Description of the Soundscape of Water Front Areas with and without Road Traffic Flow. Int. J. Environ. Res. Public Health.

[70] Gadosey C.K., Grunschel C., Kegel L.S., Schnettler T., Turhan D., Scheunemann A., Bäulke L., Thomas L., Buhlmann U., Dresel M. (2022). Study Satisfaction among University Students during the COVID-19 Pandemic: Longitudinal Development and Personal-Contextual Predictors. Front. Psychol..

[71] Wach F., Karbach J., Ruffing S., Brünken R. (2016). University Students’ Satisfaction with Their Academic Studies: Personality and Motivation Matter. Front. Psychol..

[72] Krause A.E., Scott W.G., Flynn S., Foong B., Goh K., Wake S., Miller D., Garvey D. (2021). Listening to Music to Cope with Everyday Stressors. Music. Sci..

[73] Lesiuk T. (2005). Psychology of Music The Effect of Music Listening on Work Performance. Psychol. Music..

[74] Krause A.E., Dimmock J., Rebar A.L., Jackson B. (2021). Music Listening Predicted Improved Life Satisfaction in University Students During Early Stages of the COVID-19 Pandemic. Front. Psychol..

[75] Fox J.G., Embrey E.D. (1972). Music-an Aid to Productivity. Appl. Ergon..

[76] Cockerton T., Moore S., Norman D. (1997). Cognitive Test Performance and Background Music. Percept. Mot. Ski..

[77] Ece A.S., Eren A. The Effects of Reading with Music on Reading Comprehension Global Journal on Humanites & Social Sciences. Proceedings of the 3rd World Conference On Design, Arts and Education.

[78] Kiger D.M. (1989). Music Information Load on a Reading Comprehension Task. Percept. Mot. Ski..

[79] Dosseville F., Laborde S., Scelles N. (2012). Music during Lectures: Will Students Learn Better?. Learn. Individ. Differ..

[80] Events S. Music Preferences of Teenage Students in Relation to Listener. Proceedings of the National Conference on Graduate Research in Education.

[81] Upadhyay D.D. (2013). Music Engagement, Music Preferences and Functions of Music Listening. Humanit. Soc. Sci. Stud..

[82] Nilsson M.E., Alvarsson J., Rådsten-Ekman M., Bolin K. (2010). Auditory Masking of Wanted and Unwanted Sounds in a City Park. Noise Control Eng. J..

[83] World Health Organization (2018). European Union. Environmental Noise Guidelines for the European Region.

[84] Guski R., Schreckenberg D., Schuemer R. (2017). WHO Environmental Noise Guidelines for the European Region: A Systematic Review on Environmental Noise and Annoyance. Environ. Res. Public Health.

[85] Fields J.M., De Jong R.G., Gjestland T., Flindell I.H., Job R.F.S., Kurra S., Lercher P., Vallet M., Yano T., Guski R. (2001). Standardized General-Purpose Noise Reaction Questions for Community Noise Surveys: Research and a Recommendation. J. Sound Vib..

[86] (2003). Acoustics—Assessment of Noise Annoyance by Means of Social and Socio-Acoustic Surveys.

[87] Heroux M.-E., Verbeek J. (2018). Methodology for Systematic Evidence Reviews for the WHO Environmental Noise Guidelines for the European Region. Int. J. Environ. Res. Public Health.

[88] Aletta F., Oberman T., Mitchell A., Tong H., Kang J. (2020). Assessing the Changing Urban Sound Environment during the COVID-19 Lockdown Period Using Short-Term Acoustic Measurements. Noise Mapp..

[89] Puyana-romero V., Cueto J.L., Ciaburro G., Bravo-moncayo L., Hernandez-molina R. (2022). Community Response to Noise from Hot-Spots at a Major Road in Quito (Ecuador) and Its Application for Identification and Ranking These Areas. Int. J. Environ. Res. Public Health.

[90] Keengwe J., Diteeyont W., Lawson-Body A. (2012). Student and Instructor Satisfaction with E-Learning Tools in Online Learning Environments. Int. J. Inf. Commun. Technol. Educ..

[91] Kumar P., Saxena C., Baber H. (2021). Learner-Content Interaction in e-Learning- the Moderating Role of Perceived Harm of COVID-19 in Assessing the Satisfaction of Learners. Smart Learn. Environ..

[92] Al Khattab S., Fraij F. (2011). Assessing Students’ Satisfaction with Quality of Service of Students Information System. Manag. Mark..

[93] Lipscomb S.D., Kim E.M. Perceived Match Between Visual Parameters and Auditory Correlates: An Experimental Multimedia Investigation. Proceedings of the 8th International Conference on Music Perception & Cognition.

[94] Puyana-Romero V., Maffei L., Brambilla G., Nuñez-Solano D. (2021). Sound Water Masking to Match a Waterfront Soundscape with the Users’ Expectations: The Case Study of the Seafront in Naples, Italy. Sustainability.

[95] Zelenka J. (2021). Hearing and Listening in the Context of Passivity and Activity. Open Philos..

[96] Hong J.Y., Jeon J.Y. (2015). Influence of Urban Contexts on Soundscape Perceptions: A Structural Equation Modeling Approach. Landsc. Urban Plan..

[97] Ge M., Dimopoulos I. (2003). Review and Comparison of Methods to Study the Contribution of Variables in Artificial Neural Network Models. Ecol. Model..

[98] Liddell T.M., Kruschke J.K. (2018). Analyzing Ordinal Data with Metric Models: What Could Possibly Go Wrong?. J. Exp. Soc. Psychol..

